# Semiosis as a Source of Providing Empirical Phenomena with a New Type of Cohesion

**DOI:** 10.3390/e25081173

**Published:** 2023-08-07

**Authors:** Koichiro Matsuno

**Affiliations:** Department of Bio-Engineering, Nagaoka University of Technology, Nagaoka 940-2188, Japan; cxq02365@nifty.com

**Keywords:** class property, cohesion, exchange interaction, individuality, quantum, semiosis

## Abstract

Embodying the indexical signs is vital to semiosis as a cohesive material agency mediating between consequents and antecedents. One unique factor of biology compared with standard physics and chemistry is the cohesion enabling the biological components, codes and organizations to accommodate themselves with a specific material embodiment. Every individual body is uniquely biological and requires a specific cohesion of material origin for its own sake that could not be found in the non-living material world. The relevant cohesion comes from the exchange interaction of the atomic quantum particles, such as the carbon atoms, which is far greater than the electrons as a common exchange mediator adopted for the spatial cohesion ubiquitous in physics and chemistry. What is specific to the temporal cohesion latent in the atomic exchange is the immutable identity of the individual quantum particle surviving only over a limited time, while being constantly alternated with the new ones of the same kinds in a successive manner. Semiosis is supported by the underlying teleonomic cohesion, such that the preceding temporal cohesion may constantly induce the succeeding similar one ad infinitum.

## 1. Introduction

Both polynucleotides and polypeptides, as indispensable components of biological organization, require a chemical bond in their makeup. The chemical bond based on the Heitler–London scheme, while calling attention to the exchange interaction of the orbital electrons, is about the spatial cohesion acting on the participants sharing the same present moment [1]. The spatial cohesion owes itself to the spatial confinement of the orbital electrons in a spatially closed manner, making both the events of pulling in and being pulled in simultaneous at the conceptual level.

While it has worked quite well in a wide variety of bond formations in chemical reactions underlying various biological phenomena, the spatial cohesion focused on in physics may also urge us to pay attention to one more possibility. This possibility is that the cohesion may be operative between the adjacent moments of the durable now in a spatially open manner without being subjected to the spatial confinement of the quantum particles to be exchanged. That may be the case of the temporal cohesion [2,3], in which the quantum particles to be exchanged as agents for cohesion are different to or much larger than the orbital electrons. At this point, the focus will be on the likelihood of the new cohesion that is derivable from the atomic exchange, such as the one due to exchanging the carbon atoms [4]. The exchanged atoms, spatially moving freely in both directions, to and from the environment, can also serve as the agents of the sign process. That is semiosis in both actively informing and receptively being informed of what the environment may look like towards the internal agents. The agent of semiosis can both interpret and act upon the environment, while no internal act of interpretation is allowed in the standard practice of undertaking physics.

On the other hand, the spatial cohesion upon covalent bonding is sought in the observation that the act of exchanging the orbital electron between a pair of atoms is taken to share the same individual electron at the same time in a spatially confined manner. In contrast, the temporal cohesion upon atomic exchange would be required to face the temporally cohesive nature of the atoms to be exchanged over a temporally limited, but non-zero-time interval. When temporal cohesion occurs, the stipulation of exchanging and sharing the same individual atoms rigidly at the same time in a spatially confined manner must be made somewhat looser or even further lifted in comparison with the strict case of confining the spatial cohesion locally.

When the atoms to be exchanged are found in the nearby environment, they are not so localized as in the case of the orbital electrons substantiating the covalent bonding in a spatially confined manner. Such a spatial cohesion literally in a confined manner does not apply to the atomic exchange with the environment allowing for energy dissipation through releasing the atomic components from the supporting body momentarily. Still, the cohesion derivable from the atomic exchange may survive indefinitely if the nearby environment can constantly replenish the supporting body with atoms belonging to the same class to be exchanged temporally in a tandem manner. Unique to the empirical support of semiosis is the temporal cohesion upon the exchange of individual material particles. Of course, the likelihood of temporal cohesion upon atomic exchange should have to be tested empirically, rather than simply be deduced theoretically.

## 2. Record and Action

To begin with, one likely scheme for deciphering what the temporal cohesion is all about is to shed light on how it is related to the record towards the external agent and to the action on the part of the internal agents maneuvering the cohesion. The temporal cohesion is thus about the agency relating something in retrospect to some other thing in prospect positively. The agent distinguishing prospect from retrospect is the observer internal to the phenomenon of concern, rather than the external observer. Accordingly, the temporal cohesion unique to the internal observers is agential, in contrast to the case of spatial cohesion.

The record points to what the external observer, like us, refers to as something that the internal observers possessing a synthetic competence could eventually construct and then identifies it as such externally in retrospect. In any case, the external observer is not involved in the construction of an object to be observed. The record thus understood by the external observer is about something completed so far by the internal observers, and not yet coded. In contrast, the consequential action to be figured out in reference to the record internally is totally up to the internal observers. History is constructed by each participating individual, while the consequential habit or custom may be perceived by a competent historian or the external observer not participating in the construction. What is unique to the individual is that it can experience and be experienced by the similar others in the neighborhood. However, the property of each individual per se remains unfathomable to the external observer unless it is abstracted.

On the other hand, the uniqueness of the occurrence of record rests upon its quiescence [5]. While the event registered in the present perfect tense remains quiescent, the events right in the present progressive tense can be full of agential plasticity.

Thus, a serious question may be raised with regard to how could something quiescent be accommodated with something in agential motion. The record in retrospect registered in the perfect tense remains quiescent, while the memory anchored at the record functioning as a means of anticipation in conjunction with the progressive tense to follow is agential in driving the internal observers towards the act of groping in the dark. At issue must be how we can practice our language in a congruent manner as facing the seemingly mutually incompatible empirical aspects; one is in progress and the other one is to be registered in the finished record. The matter of immediate concern here will be how the predecessor comes to be related to the successor, in which the predecessor is addressable in the present perfect tense while the successor to come proceeds in the present progressive tense.

One conspicuous remedy for the present malaise has already been available in physics in the form of the equations of motion, in which both the predecessor and the successor under the guise of being suitably abstracted are set equal to each other. However, this remedy cannot be a panacea. In order to make the equations of motion as the physical laws actually functional, they must be supplemented with the initial conditions. What must be clarified at this point is how can we guarantee the coherent unity to be contrived out of these two; one is abstract, while the other is concrete and particular in its content.

Biological organization is unique in utilizing the identity of each participating individual for the sake of securing the durable continuity of its own class’s properties exclusively in the eyes of the external observer at least phenomenologically [6]. A relevant example may be the temporal cohesion upon the atomic exchange interaction. The individual atomic identity serves as an agency for upholding the temporal cohesion for the sake of the organization even though the participating individual may be alternated with another one belonging to the same class repeatedly. One possible clue to figuring out the occurrence of temporal cohesion without relying upon the equations of motion of an abstract nature could be sought within the dynamic affinity. That affinity is already latent in the intrinsic relationship between precedents and consequents as minimizing or, if possible, as dispensing with the unwelcome intervention of an abstraction. What underlies the dynamic affinity is the temporal cohesion acting between precedents and consequents.

## 3. Relating Precedents to Consequents: A Prototypic Example of Semiosis

Relating a prior condition to its consequent as facing whatever dynamic movement is a temporal event. However, it remains open to question whether the prior condition could really be set equal to the consequent in the sense as adopted in the mechanistic scheme of the equations of motion. What is prerequisite to integrating these two in a coherent manner is the temporal cohesion of an empirical origin, rather than simply asking the mechanistic stipulation keeping the spatial cohesion intact and letting it develop temporally as riding on the rigid laws of motion [7,8].

More specifically, the citric acid cycle, in and of itself, is already indexically competent enough to trace how each of the two carbon atoms in the form of the acetyl group entering the cycle migrates inside the cycle before eventually leaving the cycle as the two carbon dioxide molecules. Accordingly, all of the carbon atoms in the cycle can be alternated with the new ones from the outside before completing the fourth round in the long run [9]. Distinguishing precedents from consequents is already latent in the natural operation of the citric acid cycle in tracing the individual historical attribute unique to each carbon atom participating there.

The radioactive labeling of carbon atoms enables us to reach at least the following three facts. Firstly, the citric acid cycle takes in two carbon atoms in the form of the acetyl group at the beginning of every round. While none of the two carbon atoms of the entering acetyl group is released from the cycle during the first round, the citric acid cycle constantly releases two carbon atoms in the form of two carbon dioxide molecules every round. Secondly, one carbon atom derivable from the entering acetyl group is released from the cycle in the form of a carbon dioxide molecule during the second round. Thirdly, the minimum number of carbon atoms constituting the carboxylic acid molecule participating in the cycle is four, while the maximum number is six. This fact that the minimum number is four is demonstrated in the four-carbon-atom carboxylic acid molecules in the cycle including succinate, fumarate, malate, and oxaloacetate [10].

These three observations, when combined, reveal that the remaining one carbon atom originating in the initial acetyl group leaves the cycle during the fourth round in the long run. This is because the oxidation of all of the pre-existing six carbon atoms in the carboxylic acid molecules in the cycle requires going round the cycle three times more at least.

Tracing the historical path of each carbon atom has been conducted on the first-in, first-out principle. It simply says that insofar as the two adjacent atoms happen to ride on the same historical path, they are in accord with keeping the rule that the later entry does not overtake the earlier one. This is no more than an expression of the irreversibility of historical development. The surviving citric acid cycle is thus a contemporaneous integration of those history-dependent carboxylic acid molecules consisting of four carbon atoms at least. Furthermore, the cycle is constantly replenished with the new carbon atoms from the outside just to compensate for those released from the cycle in the previous round, that is, to transform the preceding consequents into the succeeding antecedents.

## 4. Turning Consequents into Antecedents: Use of Contemporaneous Constellation

Sustainable operation of the citric acid cycle releasing the carbon dioxide molecules continually presumes that the cycle itself should be competent enough to replenish the carbon resources without any interruptions. That is to say, the new member carbon atom would have to be pulled into the cycle before the older incumbent member atom currently surviving in the cycle is going to leave there, rather than the older one being pulled out before the new one enters there. This is because all of the incumbent carbon atoms in the cycle, even including the older one, participate in pulling in the newest ones from the outside. Otherwise, the direct influence of the older to the newest one would be lost, unless some indexical means equivalent to guaranteeing both the memory registered in symbols and the symbol processor upon a definite coding scheme are additionally available. The sustainable citric acid cycle holds its class property intact even while each individual carbon atom constituting the cycle constantly comes and goes.

When we say a carbon atom in isolation maintains an individual identity, the agent that can recognize the identity as such must be the external observer like us. In a similar vein, when we say a carbon atom in the gas of carbon dioxide molecules maintains a common class identity, allowing for it to become part of a carbon dioxide molecule, the agent identifying the class identity as such is also the external observer. However, the agent implementing the class property out of a carbon atom through fixing and utilizing the most likely chemical affinities available internally is the immediate environment acting as the internal observer. The environment is certainly agential in demonstrating the ubiquity of quantum absorption as a local act of measurement in material terms.

Constructing the durable class identity of the products as a contemporaneous constellation of the component elements of a historical nature owes itself to the immediate environment functioning as the internal observer. The contemporaneous constellation in and of itself is teleonomic in availing the vicissitude of the individual identities to the attainment of the durable class identity, while its recognition is totally up to the external observer like us. Teleonomy is embodied simply in approaching the most likely durable pattern of chemical affinities available to the reaction environment without paying attention to the historicity of it.

Consequently, the coherent integration of both the vicissitude of the identities of the individual atoms and the persistent class identity of the reaction cycle requires some common denominator between the two. That has already been empirically confirmed in the observation that all of the carbon atoms entering the citric acid cycle have completed going round the cycle at least once. That is to say, all the contemporaneous participants have taken part in forming a durable pattern of chemical affinities as constantly letting the preceding consequents serve as the succeeding antecedents.

At this point, of course, we cannot say for sure how the memory encoded in the older incumbent atom could be decoded by the latest entry. Nonetheless, the latest entry definitely proceeds under the influence of the presence of the latest incumbents constituting the whole reaction cycle. That is teleonomic in letting the contemporaneous constellation of the incumbents set the conditions for what will arise subsequently. The successor, being dynamic in its action, is constantly formed under the influence of the quiescent predecessor stored in the record. Accordingly, this type of historical continuity connecting the dynamic action to the quiescent record must be kept since the emergence of such a reaction cycle from scratch, unless it is otherwise disturbed externally. Simply put, the durable operation of a reaction cycle may look like an age-old cliché saying that history repeats itself, while the individual participants are constantly alternated.

## 5. Class Identity and End-Directedness

One attempt for arguing for the causal power originating in the consequents may be to pay attention to the temporal sequence taken by the predecessor setting the condition for how the successor could come on the scene. Unless mechanistic stipulation is forcibly applied, there would be no likelihood such that the predecessor may exhaustively constrain all of the degrees of freedom available to the successor [11]. If there happens to be the case of making errors or any intolerable inconveniences to further be attended to, the causal power proceeding under the guise of groping in the dark will unquestionably come to generate the additional degrees of freedom to subsequently be attended to and constrained. This sequence may continue indefinitely.

Turning consequents into antecedents is thus teleonomic in the sense that something to come has already been implicated in the present moment. What is in need for naturalizing the teleonomic qualification would have to go beyond the stipulation of third person description in the present tense. Put it differently, one candidate for meeting this challenge could be to find a loophole through which one can reach third person description in the present tense as starting from second person description in the tenses other than the present tense. For third person description in the present tense is inevitable in any theoretical discourse no matter how it may be further qualified.

Telos must refer to an attribute of a specific class of causal processes. Modern biology has been upon a queer assumption that there must be an incompatibility between physicochemical characterizations of life and its teleonomic counterpart. This seemingly incompatible dichotomy could eventually be dissolved once it is recognized that the durable class identity out of a collection of the individual participants as a contemporaneous constellation of a historical nature is focused upon. The class identity can be kept even at the expense of the individual identities of the participants as allowing for constant vicissitude of the individual participants.

In other words, the class property for us as the external observer comes to impart the end-directedness or teleonomic agency to those individual participants surviving within the contemporaneous constellation, though only over a limited time in an alternating manner. Nonetheless, the individual participants may remain indifferent to what the class identity should be. The class identity appearing in a historical context available exclusively to the external observer can thus be seen as an invariant unique to the temporal development proceeding only under its unidirectional translation. The unidirectional nature of the temporal translation exhibits a marked contrast to the individual identity of an elementary particle to be kept in the reversible or bidirectional temporal translation as accepted in the standard practice of undertaking physics.

While physics is faithful to the identity of each individual, semiosis is more concerned with the identity constructed by the internal observers which is approachable to the external observer as the class identity to be coded as such. This observation suggests an inevitable discrepancy creeping in between physics and semiosis as facing the issue of what the identity of an object is. In physics, the individual identity of a physical particle has been taken as a theoretical notion of an abstract nature represented as, say, the quantum wave function. In contrast, semiosis is still open to the perspective of making the physical object called a particle something concrete enough to be directly pointed to by the participating agent without recourse to applying any abstraction. Semiosis thus requires an agent being able to maneuver a pointer. One candidate for meeting this requirement is the contemporaneous constellation of reactants, each of which carries the path-dependent history. Although it is not full-blown yet, the contemporaneous constellation serves as a legitimate substitute for what a memory is.

## 6. Instantiation of Teleonomy

What is peculiar to the end-directedness of an agency is the nature of the setter of the end-directedness itself. The setter is already an agent, whose main objective is constantly staying alive while experiencing a lot of inconveniences, troubles, or hardships. Staying alive is about durability, and the preceding durability would necessarily come to imply the succeeding similar one with use of, say, the atomic exchanges.

If something durable is derivable from those exchanges addressable in second person description, the likelihood of practicing empirical sciences anchored upon third person description may be increased. That likelihood may actually come to the surface while the external observer pays attention to the inter-agential transactions unique to the participatory observers [12,13]. This is teleonomic in the sense that the functional end constantly invites the participating agents to revisit their previous deeds and update them through interpretations, figuratively speaking. That is semiosis with no abstraction to intervene. Semiosis is in fact an instantiation of teleonomy. Self-replication, or autocatalysis, though highly abstracted in its explicit representation, is one such example. An interpretive action prerequisite to the process of semiosis as a relational agency available in the natural world, however, has to face the almost insurmountable task of addressing the issues linguistically. It has to circumvent the charge of being susceptible to anthropocentrism in a pejorative sense in one form or another.

Even unicellular organisms, such as bacteria, are the non-human sign users, which are accessible to us in second person description in the sense that we can appreciate their agential capacity with the help of a pointer available to us. In contrast, such an agential capacity on the part of the descriptive object is not permissible in a third person description in the present tense employed as a norm applied to the standard practice of performing empirical sciences. One relevant agenda must be a dependable guide to reaching functional matter as starting from inanimate matter.

## 7. From Inanimate Matter to Functional Matter

Although our primary concern is on the naturalization of functional matter that may be animate, the anthropocentric instrument for measurement may reveal at least two fundamental aspects which are also vital to the naturalized functional matter. One is the irreversibility of measurement, as implying the distinction between before and after the act of measurement. There is no way of foretelling what will be measured. The measurement of a local character could be at most a chance event only in the sense that its factual occurrence lacks pre-given global consistency. An unsettling reverberation of internal measurement may thus become inevitable.

One demonstration of the irreversibility of a naturalized measurement is revealed in the operation of the oxidative citric acid cycle proceeding in the mitochondria, rather than the reductive one. The operation of the cycle is unidirectional in that the reaction pathway from isocitrate to alpha-ketoglutarate is irreversible due to the intervention of decarboxylation as prohibiting the reversed pathway from alpha-ketoglutarate to isocitrate. The adjacent downstream reaction pathway from alpha-ketoglutarate to succinate is also irreversible because of the inevitable intervention of decarboxylation.

The likelihood of such a reaction cycle being operative in a unidirectional manner in time is certainly in conformity with the local character of measurement, in which any measurement does not presume the global consistency with the rest of the world on the spot. The act of measurement differs from the physical laws of motion, which are globally integrable, in lacking exactly this global consistency. Despite that, the external observer can read the consistent record in retrospect as dismissing any leftover from the preceding perfect tense that is vital for driving the succeeding progressive tense.

Function is normative to the functional agent in the sense that it is operative in service to the agent itself while constantly groping in the dark. For this reason, it may become fallible from time to time. A main attribute of the functional norm applied to the functional agent is to survive the occasional failures in meeting the norm. In short, the durability may be synonymous with being the most likely in an empirical sense in the eyes of the internal observers. Semiosis in action is thus in the eyes of the internal observers. Semiosis and code biology are in essence both sides of the same coin, known as the interface between the present progressive and the perfect tenses, both being referable indexically [14].

The duration of the reaction cycle is resilient, since the longer, more stable durability takes over the shorter, fragile one, if any, as the natural course of events in the process [15]. The internal observer supporting the reaction cycle is durable, at least to the extent that the cycle is durable; otherwise, the cycle would lose the indispensable supporter. One dependable scheme circumventing the present uneasiness of the internal origin is to regard the dichotomy of the self-supported and the self-supporting as the two different faces of the single agent called the self [7]. The self that is durable is constantly striving for not leaving behind any incongruent inconvenience between the supported and the supporting. What underlies the crisp enaction of self-production is the constant update of the present perfect tense in the present progressive tense for the sake of self-durability [16,17]. Here, the self is meant to be something durable on its own.

A simple example of such a functional agent as the internal observer is the citric acid cycle, which allows the individual carbon atoms to go round the cycle. It is certainly operative in service to itself. Any carbon atom constituting the cycle goes round the cycle at least once without losing its individual identity before eventually leaving the cycle. This preservation of the individual identity while going round the cycle gives the cycle the necessary cohesiveness for holding the structural identity of the cycle, which is the class identity for us as the external observer. At the same time, the carbon atoms completing the cycle at least once may be alternated with other carbon atoms without any delays. Thus, the circulating carbon atom as a sign vehicle can form a general type in its effect without sacrificing the identity of each individual atom until completing at least one turn round the cycle. The individual identity, even though kept only temporarily, is indispensable to implementing the durable class identity of the supporting reaction cycle ever since the emergence of the cycle.

The citric acid cycle, as the durable functional agent, is unique in keeping a contemporaneous constellation of the constituent carbon atoms, each of which has a unique distinctive path-dependent history behind it. What is more, the contemporaneous constellation of the carbon atoms having different histories with each other is not for the state of the citric acid cycle available to the external observer at the present moment, but only for the internal agents driving the cycle constantly forward from within.

Although it can serve as a record to the external observer like us, the contemporaneous constellation of the carbon atoms having different histories provides the functional agents as the internal observers with the guide to approaching the necessary resources in a more promising manner. Since the surviving functional agents have been successful in the preceding resource exploration, the constellation of the successful past histories may serve as a dependable and durable guide for the similar successful endeavor to come. That is teleonomic to the functional agents, which can distinguish each individual carbon atom participating in the constellation.

The durable class property is simply a consequence of the action upon the principle of first come, first served, applicable to the individual components whose identities survive only for a limited time. Functional matter comprising a reaction cycle may naturally become animate in the sense of feeding upon the resources on its own initiative when it can come to set up the contemporaneous constellation of the atomic components. Each of the components is cohesive to the others via the cycle and also carries a distinctive path-dependent history of its own.

In short, semiosis is a natural way of salvaging the durable class identity as taking advantage of the individual participants whose identities survive only over a limited time while constantly being alternated with similar others. Matter may become animate when it happens to precipitate the durable class identity out of the ephemeral individual identities. This association of animate matter with the durable class identity is, however, quite anthropocentric, since the agent that can recognize the class identity as such is limited to the external observer like us. Of course, this is not intended to denounce anthropocentrism. On the contrary, the class identity is in fact a useful linguistic vehicle for us to approach the edifice which the internal observers construct without paying attention to what the identity should be about.

The temporal cohesion upon exchanging the atomic components is quite different from the spatial cohesion upon exchanging the orbital electrons in a confined manner. In contrast, the atomic exchange is far more versatile compared with exchanging the orbital electrons in exploring the extent of cohesions. It can take advantage of the richness of the historical attributes of those atomic components constantly alternated with the new ones available from nearby. In particular, the atomic exchange comes to open a new opportunity for enhancing the extent of cohesions of material origin as utilizing the historical attributes of each atomic component participating in the exchange process.

## 8. Substrate Supporting Teleonomy

Teleonomy, we have observed, is in fact an attribute of the contemporaneous constellation of the reactants constituting a reaction cycle in which the atomic components of each reactant are constantly alternated with the new ones of the same kind. Thus, each reactant carries the path-dependent histories of the atomic components to be alternated.

This observation naturally comes to remind us that the contemporaneous constellation of the reactants to be qualified by their past histories does not meet the state description of the reaction cycle. This is because the state variables facilitating the state description acceptable in physics are path-independent. It implies that the change in the value of the state variable between the two states could be the same, even if whichever paths may be followed as starting from one of the two states until reaching another one as a destination. No irreversibility intervenes in the path-independent state description.

While accepting the state description, physics may, however, dismiss teleonomy simply on the methodological ground while doing a favor for the path-independent state description, but its dismissal has not been confirmed on the empirical ground. In order to critically examine the empirical likelihood or plausibility of teleonomy, it would be required to prepare the firm ground upon which the case of teleonomy may not have been eliminated from the start perforce. One promising strategy may be to make an appeal to empirical observations as minimizing the participation of the prior theory-laden stipulation. That is, to refer to the past history in the present addressable exclusively in concrete terms.

One advantage of referring to the contemporaneous constellation of the component elements carrying different path-dependent histories behind them is that it can address the concrete past history at the present moment of the durable now. The agential body involved is the contemporaneous constellation itself, which is in charge of constantly updating the constellation and letting the individual elements be alternated with similar ones belonging to the same class. The contemporaneous constellation as a unity is already dynamic and agential in and of itself. When it is viewed from the perspective of quantum physics, the contemporaneous constellation is agential, as revealed in the local activity of quantum absorption that is ubiquitous in the empirical world. Put simply, quantum absorption is no more than an instance of measurement of a local character.

Chemical affinity, as an indexical sign put in the status of a subject in the observational statement, may also serve as a symbol, since any subject appearing in a given sentence is supposed to stand alone. On the other hand, a similar chemical affinity as an attribute predicating the environment put in the subject turns out to be an indexical sign instead of a symbol, since it is regulated by the environment as an agential pointer [18].

If teleonomy, as the movement acting towards the end from within, is taken as a symbol, it would certainly collide against the time-honored state description accepted in physics. There is no room left for teleonomy as a symbol in the realm of physics, on the grounds that there is no symbol acting for the sake of the present in the perspective viewed from the immediate future. Any symbol admitted in physics remains quiescent in what it actually implies. However, this expelling of teleonomy from physics is not quite physical empirically. Once we are determined to face a similar issue with the use of our indexical language, a totally different perspective may arise.

Our empirical concern on durability remains incontrovertible. At this point, teleonomy enters as a scheme of salvaging a positive significance of durability empirically. The issue of immediate concern should be to shed light on whether the preservation of the individual identity of the physical body in motion could be the only allowable alternative when the likely equations of motion are descriptively approached.

One more alternative for meeting the requirement of equating consequents to precedents should be through the preservation of the class identity of the participating physical bodies in motion. Preserving the class identity allows for the individual bodies to constantly be alternated with the similar ones of the same kind. Even if the preservation of the individual identity of an object is not certain, this would not exhaustively dismiss other types of identity that may be descriptively accessible. Thus, the material substrate supporting the class identity could come to the fore [19]. It is accompanied by a new type of cohesion applied exclusively to the contemporaneous constellation of the participating individual components of an ephemeral nature that can constantly come and go. In effect, semiosis comes to put focus on the class identity, while remaining reticent on the significance of the participating individual counterpart in its descriptive endeavor [20].

## 9. Concluding Remarks

The likelihood of the physical affinity enabling the synthesis of the material substrate supporting the class identity, such as a reaction cycle, has to be examined empirically. Once the durable pattern, such as the contemporaneous constellation of reactants carrying their past histories, has been formed, it may be approached even in a third person description in the present tense as a standard norm employed for practicing empirical sciences.

However, if the durable pattern happens to experience some sway away from the durable norm, such swaying may be experienced by the functional agents as a sign assimilated to the warning to revisiting the previous fallible commitments. If a sign practiced originally in second person description has been tested as being sufficiently robust and durable, it may effectively be taken as a reliable symbol acceptable in third person description.

A sign thus assumes the double duty of pointing to both the durability almost equivalent to that referred to as a symbol and the functional agency of revisiting the efforts made for the sake of the durability so far. That semiosis is informational to the internal agent. In brief, information from within is causative.

## Data Availability

Not available.
